# Music Playlist Use in Clinical Trials of Psychedelic Assisted Psychotherapy: A Systematic Review

**DOI:** 10.1002/jclp.70167

**Published:** 2026-06-15

**Authors:** Lucas Cruz, Fernando R. Beserra, Julia M. K. Freind, Sandro E. Rodrigues, Daniel C. Mograbi

**Affiliations:** ^1^ Department of Psychology Pontifical Catholic University of Rio de Janeiro (PUC‐Rio) Rio de Janeiro Brazil; ^2^ Associação Psicodélica do Brasil (APB) Rio de Janeiro Brazil; ^3^ Institute of Psychiatry, Psychology & Neuroscience King's College London London UK

**Keywords:** music, playlist, psychedelic‐assisted psychotherapy, psychedelics

## Abstract

Recent studies have shown promising results for psychedelics in the treatment of psychiatric disorders. Both in clinical and recreational settings, contextual elements, such as music, can influence the experience of users and may be linked to positive outcomes. This systematic review identifies how music is being selected and used in psychedelisc‐assisted psychotherapy (PAP). The search adopted PRISMA criteria and was conducted using PubMed, PsycNet, Web of Science, Scopus and Cochrane as databases. The use of music in PAP was compared, considering aspects such as the creation of a playlist, playlist features and characteristics of the setting. A total of 36 articles were found, with 25 articles mentioning the use of music, predominantly addressing depressive disorders and post‐traumatic stress disorder using psilocybin and 3,4‐methylenedioxymethamphetamine (MDMA). There was considerable variation in terms of procedures, mostly because studies explored different disorders and substances, but also because there was no standardized protocol regarding music. Understanding which aspects are being privileged when choosing music for use in PAP may orient future clinical and research efforts.

## 1 Introduction

The therapeutic potential of psychedelic substances has garnered increasing scientific interest in recent years, leading to a surge in rigorous research efforts (see Yao et al. 2024; Reiff et al. 2020). Research suggests that classic psychedelics may offer novel treatment options for various mental health conditions, particularly when integrated into structured therapeutic frameworks. These substances primarily exert their effects as agonists of the serotonin 2 A receptor (5HT2A), inducing altered states of consciousness (Johnson et al. 2019). The results are promising and have drawn the attention of the scientific community (Nutt et al. 2020). Classic psychedelic substances have been used in recent clinical trials for the treatment of several psychiatric conditions, such as depression (Osório et al. 2015; Sanches et al. 2016; Palhano‐Fontes et al. 2019; Dawood Hristova and Pérez‐Jover 2023) and substance dependence (Van der Meer et al. 2023), as well as in palliative care (Gasser et al. 2015; Whinkin et al. 2023). In addition to classic psychedelics, the clinical impact of substances such as 3,4‐methylenedioxymethamphetamine (MDMA), which can be termed an empathogen, has also been investigated (Fonseka and Woo 2023). Currently, clinical research with MDMA is in phase 3, with significant results in the treatment of PTSD (Mitchell et al. 2021; Smith et al. 2022).

The therapeutic use of psychedelics diverges from current pharmacotherapy models because, for reasons of safety (Romeo et al. 2024) and possibly efficacy (Krupitsky and Grinenko 1997; Cavarra et al. 2022), it requires the use of these substances to be conducted within a supportive psychotherapeutic context. The association between psychotherapy support and psychedelics has led to the term “psychedelic‐assisted psychotherapy” (PAP). This approach stems from the notion that the therapeutic effects of psychedelics are context‐dependent (Hartogsohn 2017; Carhart‐Harris et al. 2018). The psychedelic experience and its effects are directly influenced by three interrelated dimensions: set, referring to individual aspects, such as intentions, expectations, and physical and psychological state before the experience; setting, encompassing the environment in which the session occurs; and matrix (Eisner 1997), which represents the broader framework surrounding psychedelic sessions, including the cultural and economic aspects of the environment from which an individual comes and to which the individual returns after a session (Rodrigues 2024). Music is one of the most discussed elements of the therapeutic setting in PAP, although there is a need for more empirical research (Golden et al. 2022). Choosing the musical setting carefully when using psychedelics is a longstanding practice and a recurrent feature in traditional use, also remaining relevant in psychedelic psychotherapy approaches in the 1950s and 60 s (Kaelen et al. 2018). For example, Bonny and Pahnke (1972) proposed a model to structure psychedelic experiences, highlighting the role of music as a key factor in shaping the trajectory of the session, dividing it into “pre‐onset”, “onset”, “building towards peak intensity”, “peak intensity of drug action”, “re‐entry”, and “return to normal consciousness”.

Similarly, in contemporary protocols used in clinical research with psychedelics, one of the central elements of the setting during sessions is the use of music (Mithoefer 2015; Guss et al. 2020). Music has been shown to enhance emotional and cognitive responses to psychedelics, contributing to the overall therapeutic process (Preller et al. 2017; Watts et al. 2017). Evidence suggests that music not only amplifies emotional depth but also helps individuals confront and process difficult emotions. For instance, qualitative analyses of patients treated with psilocybin‐assisted therapy for treatment‐resistant depression indicate that music facilitated emotional surrender and the emergence of repressed feelings, often guiding participants through challenging but therapeutically meaningful experiences (Watts et al. 2017). Music is relevant in PAP due to its possible ability to help individuals work through experiences with therapeutic importance, intensify emotions, and enhance mental imagery. Additionally, music has been linked to the emergence of mystical‐type experiences and insightfulness, both of which contribute to the therapeutic benefits of PAP (Kaelen et al. 2018), based on a qualitative phenomenological study with exploratory correlational analyses. In this context, allowing participants to select music may contribute to a greater sense of familiarity and security toward the end of the experience (O'Callaghan et al. 2020).

While music is widely recognized as an important element in PAP (Kaelen et al. 2018; Barrett et al. 2018), research on how to structure and select music remains limited. Recent efforts have sought to address this issue. Messell et al. (2022) proposed a structured protocol for music use in PAP, aligning musical intensity with psychedelic effects to possibly enhance therapeutic outcomes. This approach draws on Guided Imagery and Music (Bonny 2002) and emphasizes the need for standardization in therapy. Although this framework is a significant step forward, further research is needed to develop standardized methods for playlist construction and evaluate their impact on therapeutic results. Considering the above, this review aims to identify, characterize, and evaluate the use of music and playlists in clinical applications of classic psychedelics and MDMA.

## 2 Methods

A systematic review was conducted exploring the use of music in PAP. The systematic review was registered on the International Prospective Register of Systematic Reviews (PROSPERO) under registration number CRD42022365423. PRISMA recommendations were followed (Page et al. 2021). Search results were imported into Rayyan, a licensed website designed to assist authors in conducting systematic reviews. This research platform facilitates collating articles, identifying reasons for article exclusion, and tracking decisions, with independent assessments made by each reviewer to reduce potential bias (Ouzzani et al. 2016). Articles were then assessed according to the eligibility criteria, after which two independent researchers (L.C. & J.F.), who were unaware of each other's evaluations, reviewed titles and abstracts. After this stage, reviewers compared their decisions and discussed any discrepancies. In instances where uncertainties regarding the criteria arose, a third reviewer was consulted (F. B).

Studies were categorized according to features such as method, sample, substance used, and setting, and regarding music playlist characteristics, such as its source, number of songs, music genres, length, whether it considers the phases of psychedelic experience and whether it has vocals.

**Figure 1:**
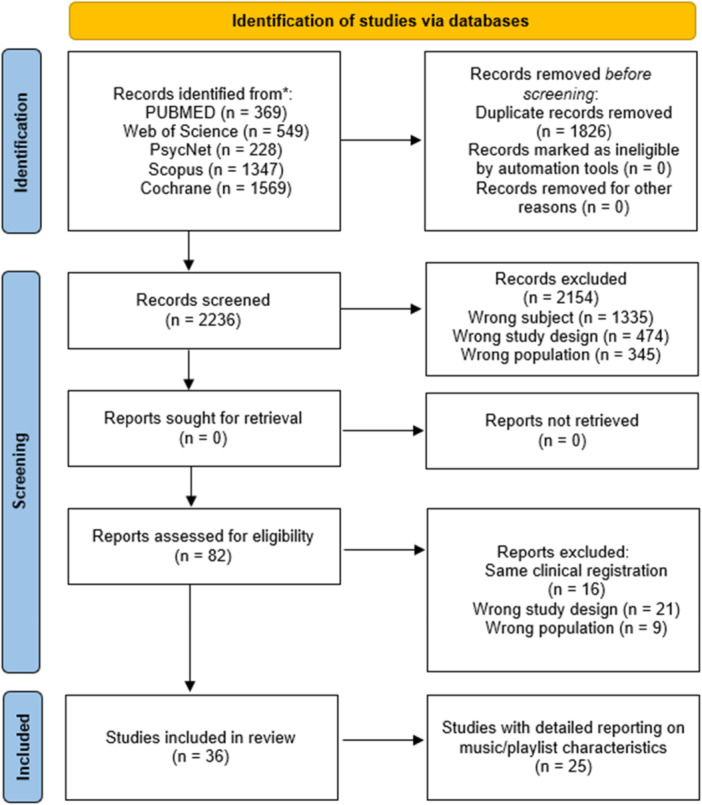
PRISMA flowchart. *Total of articles was 4062.

The procedures of the review can be seen in Figure 1.

### 2.1 Study Selection Criteria

Clinical research was included in the review if it focused on the treatment of one or more psychiatric disorders with the use of psychedelic substances, including classic psychedelics (LSD, psilocybin, ayahuasca/dimethyltryptamine [DMT], and mescaline) and MDMA. The following study designs were included: randomized clinical trials (RCT) and open‐label studies without placebo control. Observational studies, case studies and review articles were not included. Studies that focused on other substances or methods were also excluded. The search initially covered a 10‐year period. This coincides with an increase in the number of articles using psychedelic interventions, also providing a focus on more recent evidence. The timeframe was later extended to ensure the inclusion of the most recent studies available by the time of the review's completion. As a result, research published between January 2013 and May 2024 was considered. Given the multitude of studies on MDMA‐assisted psychotherapy available in the literature, the inclusion of these articles was deemed relevant to broaden the scope of this systematic review. Only articles written in English were included.

Our focus was on the music used in clinical trial studies. For this reason, the search strategy was developed to maximize the number of clinical trial studies retrieved. It would deviate considerably from our aims to explore music use in general in psychedelic research.

### 2.2 Search Strategy

The search was conducted in PubMed, Web of Science, PsycNet, Scopus, and Cochrane platforms. These databases are among the most used and have considerable overlap with others. Keywords employed are listed in the supplementary material, with all terms verified through MeSH. Finally, relevant information about music (e.g., length, genres, number of songs, languages) was gathered from articles, supplementary materials, or multiple sources. When not explicitly stated, information about the music used in the intervention was usually obtained from ClinicalTrials. gov, a comprehensive database of clinical studies and their outcomes, or, in some cases, through direct contact with authors.

### 2.3 Quality Assessment

The quality assessment was performed using the scoring system presented in Kmet et al. (2004). This scoring system provides a systematic, reproducible and quantitative means of assessing the quality of research. The chosen criteria can be seen in Table 1. The assessment was performed by two independent reviewers (L.C. & J.F.). 14 aspects were scored as “yes” (2 points), “partial” (1 point) or “no” (0 points). A summary score was calculated for each paper by dividing the article's total score by the total possible score (28). If both reviewers agreed that the items had a summary score below 0.75 in the quality assessment, the paper was excluded from the review. If there was not a consensus, the study was submitted for evaluation by a third independent reviewer (F.B.). In this study, none of the papers were excluded because of the quality assessment and there was no disagreement between the reviewers about these exclusions.

**Table 1:** Quality Assessment Criteria.

Criteria	Yes	Partial	No
Question/objective sufficiently described?			
Study design evident and appropriate?			
Method of subject/comparison group selection or source of information/input variables described and appropriate?			
Subject (and comparison group, if applicable) characteristics sufficiently described?			
If interventional and random allocation was possible, was it described?			
If interventional and blinding of investigators was possible, was it reported?			
If interventional and blinding of subjects was possible, was it reported?			
Outcome and (if applicable) exposure measure(s) well defined and robust to measurement/misclassification bias? Means of assessment reported?			
Sample size appropriate?			
Analytic methods described/justified and appropriate?			
Some estimate of variance is reported for the main results?			
Controlled for confounding?			
Results reported in sufficient detail?			
Conclusions supported by the results?			

## 3 Results

The PRISMA flowchart can be seen in Figure 1. A total of 369, 228, 549, 1347, and 1569 papers were identified, respectively, in PubMed, PsycNet, Web of Science, Scopus, and Cochrane, totaling 4062 articles for initial title and abstract screening. 1826 records were removed before screening because they were duplicates. After a review of titles and abstracts, 2154 articles did not meet the eligibility criteria and were also excluded from the analysis, leaving 82 full‐text articles for further screening. Among these studies, 46 were excluded due to not conforming to the selected study designs, absence of a clinical population or because they had the same clinical registration as other articles already analyzed. In the end, 36 articles were included in our review. A detailed description of each study and its playlist features is provided in the Supporting Material (S1).

### 3.1 Sample

Regarding the sample, articles investigated different disorders: depressive disorders (15), post‐traumatic stress disorder (8), anxiety disorders (3), alcohol dependence (2), tobacco addiction (1), anxiety and depression for life‐threatening disease (5), anorexia nervosa (1), and body dysmorphic disorder (1).

### 3.2 Study Design

Regarding the methods, 20 studies employed a randomized controlled trial design, 13 were open‐label and the other 3 studies used both methods. One of the articles contained two studies, one being a randomized clinical trial, and the other an open‐label study (Daws et al. 2022). Additionally, there was one exploratory, placebo‐controlled, fixed‐order trial (Sloshower et al. 2023).

### 3.3 Psychedelic Substance

Of the 36 articles included, 18 used psilocybin, 1 compared psilocybin with escitalopram, 11 investigated the effects of MDMA, 5 of ayahuasca/DMT and 1 of LSD.

### 3.4 Use of Music

25 articles stated that they used at least one music playlist, 8 did not mention music and 3 explicitly affirmed it was not used. Of the latter 3, one justified not using music with the intention of investigating solely the pharmacological effects of ayahuasca, with the exclusion of non‐drug factors commonly related to religious rituals (Sanches et al. 2016). The other study indicated that music was not used because the goal was to have a setting as quiet and neutral a space as possible (Dos Santos et al. 2021). One study did not justify the absence of music (D'Souza et al. 2022). Despite the lack of music, all three studies reported positive therapeutic outcomes, suggesting that while music can serve as an important facilitator of emotional and experiential depth, its presence may not be strictly necessary for the occurrence of beneficial effects.

Between the 25 studies which used a playlist,13 of them provided more details about the music through email or in their own article, and 7 even provided a link to the playlist used. At least 10 of the playlists considered the phases of the psychedelic experience, as confirmed by email. Notably, some playlists were used in multiple studies. The Johns Hopkins University playlist was used in two studies (Davis et al. 2021; Rosenblat et al. 2024), while Kaelen's playlists were employed in Murphy et al. (2022), Sloshower et al. (2023), and Sessa et al. (2021), with the latter reporting the use of more than one Kaelen playlist. These playlists thus appear to be the most frequently utilized across the included studies. Both playlists have been included in the reference list.

About the source of the playlist, 9 articles mentioned using streaming services. In one study they used CDs and iTunes. Regarding the length of the playlist, 14 studies provided this information—either directly in the article, through email correspondence, or via the playlist link—showing a variation between approximately 1.5 and 7.3 h. The number of songs varied between 6 and 98 songs in each playlist, but most (*n* = 6) of the playlists contained between 40 and 70 songs. The mean duration of the playlists was approximately 333.7 min (around 5 h and 34 min), with a standard deviation of 116 min (approximately 1 h and 56 min).

10 articles stated that they used music with vocals in their playlists. One of them added that they did not use lyrics with negative or destructive messages (Oehen et al. 2013) and another study highlighted that, for the most part of the playlist, they tried to use words that are not in a language which the participant could understand (Ot'alora G et al. 2018). Additionally, one author, when asked via email, emphasized that lyrics should be strictly avoided in psychedelic therapy playlists. One study used 2 playlists: one with and another without lyrics (Palhano‐Fontes et al. 2019). Another study used five playlists provided by Kaelen (Sessa et al. 2021); according to the author (personal communication by email), the team would select or switch between playlists depending on the psychological state of the patient during the session. Musical genres were diverse, but there was mostly the presence of classical music, new age, jazz, opera and ambient music. A name was provided for only seven playlists.

Additionally, one of the studies provided a hyperlink for posterior use by research participants to support their work in integrating contents, reconnecting and remembering meaningful aspects of their experience. To create the playlist, they also consulted with various therapists, musicians, imaging experts and psychedelic elders (Shnayder et al. 2023). In one of the studies, participants were also given the option to add three personal music choices to play at the end of the session (Murphy et al. 2022).

### 3.5 Setting

34 studies mentioned directly the presence of guides (which include therapists, psychiatrists or facilitators). 2 studies did not mention any information about guides or the setting itself. 8 of the studies mentioned the physical space but did not describe in detail the environment in which the substance was administered, informing only where it happened (e.g., outpatient clinic). 14 studies showed a concern about pre‐decorating the place, making it comfortable and promoting a calm atmosphere. In 20 studies, participants were stimulated to use eyeshades or close their eyes. Regarding delivery of music, 13 of the studies cited headphones, and two of them also mentioned the use of speakers and earphones or headphones. (Carhart‐Harris et al. 2016). In one of the studies, therapeutic sessions were conducted in groups (Oehen et al. 2013) Table 2.

**Table 2:** Summary of Reported Features in the Included Studies.

Feature	Number of studies reporting
Studies reporting the use of playlists	25
Studies considering psychedelic experience phases in playlists	10
Playlist details (length, source, genres, etc.)*	13
Guides/protocols reported	34
Study setting details reported	14
Eyeshades used	20
Headphones used	13

* For a more detailed description of playlist characteristics, see S1.

## 4 Discussion

This study aimed to evaluate the current use of music, a significant aspect of PAP. In this systematic review, we found that despite the high importance attributed to music in psychedelic research, there are few records on the specific role of music in PAP or its possible effects on clinical outcomes. Additionally, information about the creation of playlists and the presence of music therapy professionals during the creation or playback of these playlists is scarce. We also observed that not all articles use playlists in a structured manner, often leaving the decision about the presence or absence of music to the research participants during therapeutic sessions. Depression and PTSD were the most frequently investigated conditions, reflecting broader trends in psychedelic clinical research.

There is a predominant understanding among psychedelic researchers that music is a relevant component of the therapeutic setting, whether for safety reasons and/or for the establishment of a more effective clinical protocol, for instance providing psychological support, grounding, reassurance, and a sense of continuity and direction throughout the session (Kaelen et al. 2018). In the current review, most clinical studies used music playlists, with only three studies stating that no music was used. While music has long been considered central to psychedelic therapy, our findings highlight variability in how consistently it is implemented and reported in contemporary trials.

Ayahuasca/DMT stands out as the substance most frequently investigated without explicit music use, whereas psilocybin trials almost uniformly incorporated playlists. The investigation of the effects of ayahuasca/DMT without music is striking, considering its ritualistic use, both by indigenous people from the Amazon region and syncretic traditions, such as Santo Daime, that often rely on music and chanting as part of ceremonies.

Although phase‐based structuring remains influential in the development of playlists (e.g. Mithoefer 2015), most contemporary trials do not clearly report whether or how these phases informed playlist construction. Although this could be tracked in the case of a few studies by contacting authors by e‐mail or following the reference to the playlist development, an important recommendation for future clinical trials is to explicitly state the structuring of the playlist in relation to phases of the psychedelic experience. This may be tailored in terms of time according to the duration of effects depending on dose and substance, but a consistent structure may enhance comparability of future studies.

The duration of playlists varied considerably, generally aligning with the expected duration of acute drug effects across substances. Some studies, such as Oehen et al. (2013), allowed participants to choose whether to listen to music, raising the question of whether non‐standardized music use affects clinical outcomes. Future research should balance protocol standardization—which facilitates methodological replication—with the need for participant comfort and autonomy. This tension between standardization and personalization represents a central empirical question for future research, particularly regarding its impact on safety, emotional regulation, and clinical outcomes.

**Figure 2:**
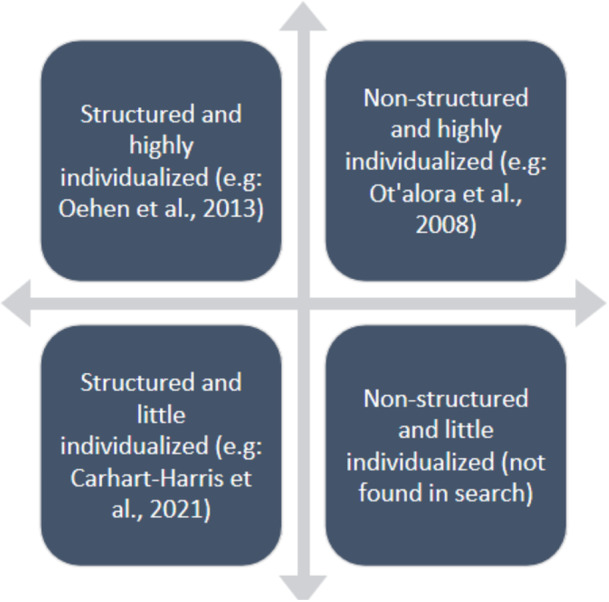
Playlist Models Use in Psychedelic Clinical Research.

In the research reviewed, playlists used were organized in different ways, with more or less structured models and with greater or lesser attention to the individuality of the research participant. Figure 2 aims to cover the diversity of ways playlists can be used in clinical research with psychedelics, where the reviewed studies fall in this matrix. Attention to participants' individuality can come in the form of autonomy to select songs for the playlist or the playlist author himself considering participants' tastes and preferences. Structured playlists consider not only the use of the same material for all participants but also the presence of criteria for the playlist composition.

One potential compromise is indicated by Murphy et al. (2022), in which participants were invited to include three songs of their personal choice at the end of the session. Perhaps a crucial distinction should be made between research protocols, which should be as standardized as possible, and clinical practice, in which patients may benefit from a personalized approach. Ultimately, an important question for future research is which approach—structured or individualized—leads to better clinical outcomes, highlighting the need for empirical comparisons between these models.

Grof (1980) recommended using music with vocals in unfamiliar languages to avoid influencing the psychedelic experience. Given psychedelics' potential to enhance suggestibility (Dupuis and Veissière 2022), the choice between instrumental and vocal music remains a relevant consideration for future research. Moreover, it is possible that musical effects depend on specific musical parameters such as tempo, timbre, variability, and instrumentation. More research exploring these variables would be valuable to better understand their potential influence on participants' experiences and therapeutic outcomes.

Rodrigues (2021) suggests that there are no good or bad musical genres per se, but that their effects depend on their particular use for each person in each context. In the context of standardized research protocols, genres with more universal exposure, such as classical music, may be particularly useful. This should be balanced with culture sensitivity and use of local music that is meaningful to participants.

In the studies evaluated, most explicitly mentioned encouraging participants to wear eyeshades or to close their eyes. Reducing visual input may enhance the experience of music, highlighting the importance of this dimension during sessions. Another aspect to be considered when thinking about music in the setting of psychedelic experiences is sound quality, so it is important to carefully choose headphones, sound systems, and quality of files (Kaelen 2021).

This review presented a comprehensive and current synthesis of the use of music in PAP, contributing to the literature by systematically mapping playlist utilization across diverse clinical trials. Notably, the inclusion of supporting materials and direct correspondence with authors allowed for the retrieval of contextual information often unavailable in published reports, thereby enriching the analysis and enhancing the interpretative depth of the findings. The limitations of our study include a scarcity of extensive prior literature on the subject and limited data availability. Randomized clinical trials and open‐label studies often prioritize reporting other information, and we hope that this review may highlight the importance of music in this type of research.

Future research may benefit from being organized around three central directions. First, there is a need for study designs capable of more clearly testing music's specific contribution to clinical outcomes in psychedelic‐assisted psychotherapy, including comparative designs that manipulate the presence, structure, or degree of personalization of playlists. Such approaches would help clarify whether structured, individualized, or hybrid models differentially affect safety, emotional processing, and therapeutic efficacy. Second, future studies should examine specific musical parameters, such as tempo, timbre, variability, instrumentation, and the presence or absence of lyrics, in order to better understand how particular acoustic features interact with psychedelic states and influence subjective experience and clinical response. Third, improved reporting practices are essential to enhance transparency and comparability across trials. We recommend that future studies systematically describe playlist construction, duration, sequencing rationale, delivery methods, and sound equipment, ideally using standardized extraction tables such as the model provided in this review. Greater methodological clarity will facilitate cumulative knowledge building in this emerging field.

## 5 Conclusions

This systematic review outlined the utilization of playlists in PAP aimed at treating mental disorders. While most articles declare that they use playlists, only a few provide specific details about their composition. A broad spectrum of musical genres was identified, encompassing both vocal and instrumental selections. Few articles explicitly mention considering the different phases of the psychedelic experience when developing the playlists. Despite the apparent significance of musical support in PAP, there is limited adherence to recommendations on playlist design. Notably, professionals in music therapy are seldom referenced in the playlist creation process, and descriptions of the sound quality/equipment were also missing in the articles found. A framework was devised to categorize and conceptualize playlists, based on their structure (structured/unstructured) and the degree of individualization (highly individualized/poorly individualized). It has been suggested that research protocols may veer towards a more structured approach, with clinical care potentially being more effective with personalized material.

## Supporting information

Supporting File 1

Supporting File 2

Supporting File 3

## Data Availability

The data that support the findings of this study are available in the supporting material of this article.
