# Supplementary material for: Music Playlist Use in Clinical Trials of Psychedelic Assisted Psychotherapy: A Systematic Review
Source: J Clin Psychol. 2026 Jun 15;82(9):1263–70. doi: 10.1002/jclp.70167 (PMC13441328; doi:10.1002/jclp.70167)
Supplement: Supplementary file 1 [file JCLP-82-1263-s001.docx]

| **Authors and year** | **Method** | **Sample** | **Drug** | **Does the research use music playlist?** | **Does the playlist take in account the phases of psychedelic experience?** | **Which is the length of the playlist?** | **Music genres** | **Musics have vocal?** | **Number of songs** | **Playlist name** | **Playlist Source** | **Playlist models use in psychedelic clinical research** | **Sitter** | **Eyeshade** | **Headphone** |
| --- | --- | --- | --- | --- | --- | --- | --- | --- | --- | --- | --- | --- | --- | --- | --- |
| Bogenschutz et al. (2022) | RCT + Open-label | 95 participants with alcohol dependence | Psilocybin | Yes | -- | -- | -- | -- | -- | -- | -- | Unkown | Yes | Yes | Yes |
| Carhart-Harris et al. (2021) | RCT | 59 patients with moderate-to-severe MDD | Psilocybin | Yes | Yes | 7h21 | Classical (Indian and Western) and ambient music | Yes | 68 | Psilodep Session 2 | Spotify | Structured and litlle individualized | Yes | Yes | Yes |
| Carhart-Harris et al. (2016) | Open-label | 12 patients with moderate-to-severe TRD | Psilocybin | Yes | Yes | Approx 6h | Various genres such as opera, new age and jazz | Yes | 8 | Playlist (Psilocybin for depression, Imperial College London, version 1.3) | Mixcloud | Structured and litlle individualized | Yes | Yes | Speakers + Earphones |
| Danforth et al. (2018) | RCT | 12 autistic adults with marked to very severe SAD | MDMA | -- | -- | -- | -- | -- | -- | -- | -- | Unkown | -- | -- | -- |
| Davis et al. (2021) | RCT | 24 participants with MDD | Psilocybin | Yes | Yes | 5h30 | Classical, New Age, Jazz | Yes | 49 | -- | Spotify | Structured and litlle individualized | Yes | Yes | Yes |
| Daws et al. (2022) | RCT + Open-label | Study 1: 19 participants with TRD; Study 2: 59 participants with MDD | Psilocybin or escitalopram | -- | -- | -- | -- | -- | -- | -- | -- | Unkown | -- | -- | -- |
| Dos Santos et al. (2021) | RCT | 17 patients with SAD | Ayahuasca | No | No | No | No | No | No | No | No | No Music | Yes | No | No |
| Gasser et al. (2014) | RCT | 12 patients with anxiety associated with life-threatening diseases | Ayahuasca | Yes | -- | -- | -- | -- | -- | -- | -- | Unkown | Yes | -- | -- |
| Goodwin et al. (2022) | RCT | 233 patients with TRD | Psilocybin | Yes | Yes | 7 hours | Wide range of genres including avant-garde, jazz, neo classical, new age, rock. | Only 2 at the very end of the playlist | 61 | -- | Created in tidal high resolution streaming | Structured and litlle individualized | Yes | Yes | -- |
| Griffiths et al. (2016) | RCT | 56 participants with a potentially life-threatening cancer diagnosis + anxiety and/or mood symptoms | Psilocybin | Yes | -- | -- | -- | -- | -- | -- | -- | Unkown | Yes | Yes | Yes |
| Holze et al. (2023) | RCT | 42 patients with anxiety with/without association with a life-threatening illness | LSD | Yes | -- | -- | -- | -- | -- | -- | -- | Unkown | Yes | -- | -- |
| Jardim et al. (2021) | Open-label | 6 patients with PTSD secondary to sexual abuse | MDMA | Yes | -- | -- | -- | -- | -- | -- | -- | Unkown | Yes | -- | -- |
| Johnson et al. (2014) | Open-label | 15 participants (smokers) | Psilocybin | Yes | -- | -- | -- | -- | -- | -- | -- | Unkown | Yes | Yes | -- |
| Mitchell et al. (2021) | RCT | 90 patients with PTSD | MDMA | -- | -- | -- | -- | -- | -- | -- | -- | Unkown | Yes | -- | -- |
| Mithoerfer et al. (2018) | RCT | 26 patients with PTSD | MDMA | -- | -- | -- | -- | -- | -- | -- | -- | Unkown | Yes | -- | -- |
| Monson et al. (2020) | Open-label | 12 participants. 6 couples (at least 1 per couple with PTSD) | MDMA | -- | -- | -- | -- | -- | -- | -- | -- | Unkown | Yes | -- | -- |
| Murphy et al. (2022) | RCT | 59 patients with moderate to severe MDD | Psilocybin | Yes | Yes | 5h30 | Classical, New Age, Jazz, EDM... | Yes | 49 | Psychedelic Therapy Playlist 1 | Spotify | Structured and litlle individualized | Yes | Yes | Yes |
| Oehen et al. (2013) | RCT | 12 patients with TR-PTSD | MDMA | Yes | Yes | 1.5-3h/8h session depending on the individual need for musical support | All kind of music genres. | Yes. No lyrics with negative or destructive messages. | 15 - 25 pieces of music (depending on the lenght) | -- | CDs and iTunes | Structured and highly individualized | Yes | Eyes closed | -- |
| Ot'alora et al. (2008) | RCT | 28 patients with PTSD | MDMA | Yes. Study used around 20 different playlists | Yes | playlist are 8 to 10 hours long | -- | Mostly instrumental. A few songs have words. | Varies within each playlist but it is between 8-10 hours long | -- | Spotify | Non-structured and highly individualized | Yes | Yes | Yes |
| Palhano-Fontes et al. (2019) | RCT | 29 patients with TRD | Ayahuasca | Yes | Yes | Aprox 4h | Brazilian singers, instrumental, classic and andean music | Yes. | 1 (with lyrics): 98 songs \| 2 (instrumental): 24 songs | Ayahuasca therapeutic playlist (with lyrics)\|Ayahuasca therapeutic playlist (instrumental) | Spotify | Structured and litlle individualized | Yes | Eyes closed | -- |
| Ross et al. (2016) | RCT | 29 patients with cancer-related anxiety and depression | Psilocybin | -- | -- | -- | -- | -- | -- | -- | -- | Unkown | Yes | -- | -- |
| Sanches et al. (2016) | Open-label | 17 patients with recurrent MDD | Ayahuasca | No | N/A | N/A | N/A | N/A | N/A | N/A | N/A | No Music | Yes | -- | -- |
| Shnayder et al. (2022) | Open label | 30 patients with cancer associated MDD | Psilocybin | Yes | Yes | Aprox 9h | Various. Mostly classic music, | Some | 63 | -- | Sunstone Therapies website | Unkown | Yes | Yes | -- |
| Von Rotz et al. (2022) | RCT | 52 patients with MDD | Psilocybin | Yes | -- | -- | -- | -- | -- | -- | -- | Unkown | Yes | -- | Headphones or speakers |
| Wang et al. (2021) | Open-label | 37 patients with PTSD | MDMA | -- | -- | -- | -- | -- | -- | -- | -- | Unkown | Yes | -- | -- |
| Wolfson et al. (2020) | RCT + Open-label | 18 patients with anxiety related to LTI | MDMA | Yes | -- | -- | -- | -- | -- | -- | -- | Unkown | Yes | Yes | Yes |
| Goodwin (2023) | Open-label | 19 TRD patients | Psilocybin | Yes | -- | -- | -- | -- | -- | -- | -- | Unkown | Yes | Yes | Yes |
| Mitchell et al. (2023) | RCT | PTSD | MDMA | Yes | **--** | -- | -- | -- | -- | -- | -- | Unkown | Yes | Yes | Yes |
| Peck et al. (2023) | Open-label | 10 females with anorexia nervosa | Psilocybin | -- | -- | -- | -- | -- | -- | -- | -- | Unkown | Yes | Yes | Yes |
| Schneier et al. (2023) | Open-label | Body dysmorphic disorder | Psilocybin | Yes | -- | -- | -- | -- | -- | -- | -- | Unkown | Yes | Yes | -- |
| Sloshower et al. (2023) | RCT | MDD | Psilocybin | Yes | Yes | 5h30 | Classical, New Age, Jazz, EDM... | Yes | 49 | Psychedelic Therapy Playlist 1 | Spotify | Structured and litlle individualized | Yes | Yes | Yes |
| Rosenblat et al. (2024) | RCT | 30 patients with TRD | Psilocybin | Yes | Yes | 5h30 | Classical, New Age, Jazz... | Yes | 49 | -- | Spotify | Structured and litlle individualized | Yes | -- | -- |
| Raison et al. (2023) | RCT | 104 patients with MDD | Psilocybin | Yes | No | About 8h | Mostly "new age" or "world music" with some classical | No | No info | No info | Spotify | Unkown | Yes | Yes | Yes |
| D'Souza et al. (2022) | Open-label | 7 patients with MDD | DMT | No | N/A | N/A | N/A | N/A | N/A | N/A | N/A | No Music | Yes | -- | No |
| Sessa et al. (2021) | Open-label | 14 patients with AUD | MDMA | Yes | Yes | Varied. | Ambient chill. Some world music. | Yes | 28 (sum of playlists songs) | Kaelen playlists (more than one) | Spotify | Structured and litlle individualized | Yes | -- | -- |
| Lewis et al. (2023) | Open-label | 12 patients | Psilocybin | Yes | Yes | 7 h 30 min | Classical, New Age, Devotional Music | Yes | 61 | U of U Psychedelic-Assisted Psychotherapy Playlist | Spotify | Unkown | Yes | -- | -- |
