# Supplementary material for: Music Playlist Use in Clinical Trials of Psychedelic Assisted Psychotherapy: A Systematic Review
Source: J Clin Psychol. 2026 Jun 15;82(9):1263–70. doi: 10.1002/jclp.70167 (PMC13441328; doi:10.1002/jclp.70167)
Supplement: Supplementary file 2 [file JCLP-82-1263-s002.docx]

**Search Strategy**

**PUBMED**:

Psilocybin*[TIAB] OR DMT[TIAB] OR N Methyl 3,4 methylenedioxyamphetamine[TIAB] OR MDMA[TIAB] OR Methylenedioxymethamphetamine[TIAB] OR Ecstasy[TIAB] OR N-Methyl-3,4-methylenedioxyamphetamine Hydrochloride[TIAB] OR Hydrochloride, N-Methyl-3,4-methylenedioxyamphetamine[TIAB] OR N Methyl 3,4 methylenedioxyamphetamine Hydrochloride[TIAB] OR N,N-Dimethyltryptamine[TIAB] OR Banisteriops*[TIAB] OR Ayahuasca*[TIAB] OR LSD[TIAB] OR LSD-25[TIAB] OR LSD 25[TIAB] OR Lysergic Acid Diethylamide[TIAB] OR Acid Diethylamide, Lysergic[TIAB] OR Diethylamide, Lysergic Acid[TIAB] OR Lysergide[TIAB] OR Lysergic Acid Diethylamide Tartrate[TIAB] OR Mescaline[TIAB] OR Mezcalin[TIAB] OR Peyote[TIAB] OR Trimethoxyphenethylamine [TIAB] OR 3,4,5-Trimethoxyphenethylamine[TIAB] AND ((y_10[Filter]) AND (clinicaltrial[Filter] OR randomizedcontrolledtrial[Filter])) AND ((y_10[Filter]) AND (clinicaltrial[Filter] OR randomizedcontrolledtrial[Filter]))

*Time filter publication was January 2013 to May 2024.

**Web of Science:**

((((((((((((((((((((((((((((AB=(Psilocybin)) OR AB=(Psilocybine)) OR AB=(DMT)) OR AB=(N Methyl 3,4 methylenedioxyamphetamine)) OR AB=(MDMA)) OR AB=(Methylenedioxymethamphetamine)) OR AB=(Ecstasy)) OR AB=(N-Methyl-3,4-methylenedioxyamphetamine Hydrochloride)) OR AB=(Hydrochloride, N-Methyl-3,4-methylenedioxyamphetamine)) OR AB=(N Methyl 3,4 methylenedioxyamphetamine Hydrochloride)) OR AB=(N,N-Dimethyltryptamine)) OR AB=(Ayahuascas)) OR AB=(Banisteriopses)) OR AB=(Banisteriopsis)) OR AB=(Ayahuasca)) OR AB=(LSD)) OR AB=(LSD-25)) OR AB=(LSD 25)) OR AB=(Lysergic Acid Diethylamide)) OR AB=(Acid Diethylamide, Lysergic)) OR AB=(Diethylamide, Lysergic Acid)) OR AB=(Lysergide)) OR AB=(Lysergic Acid Diethylamide Tartrate)) OR AB=(Mescaline)) OR AB=(Mezcalin)) OR AB=(Peyote)) OR AB=(Trimethoxyphenethylamine)) OR AB=(3,4,5-Trimethoxyphenethylamine)) AND AB=(clinical trial)

*Time filter publication was January 2013 to May 2024.

**PsycNet:**

**Abstract**: Psilocybin *OR* **Abstract**: Psilocybine *OR* **Abstract**: DMT *OR* **Abstract**: N Methyl 3,4 methylenedioxyamphetamine *OR* **Abstract**: MDMA *OR* **Abstract**: Methylenedioxymethamphetamine *OR* **Abstract**: Ecstasy *OR* **Abstract**: N-Methyl-3,4-methylenedioxyamphetamine Hydrochloride *OR* **Abstract**: Hydrochloride, N-Methyl-3,4-methylenedioxyamphetamine *OR* **Abstract**: N Methyl 3,4 methylenedioxyamphetamine Hydrochloride *OR* **Abstract**: N,N-Dimethyltryptamine *OR* **Abstract**: Ayahuascas *OR* **Abstract**: Banisteriopses *OR* **Abstract**: Banisteriopsis *OR* **Abstract**: Ayahuasca *OR* **Abstract**: LSD *OR* **Abstract**: LSD-25 *OR* **Abstract**: LSD 25 *OR* **Abstract**: Lysergic Acid Diethylamide *OR* **Abstract**: Acid Diethylamide, Lysergic *OR* **Abstract**: Diethylamide, Lysergic Acid *OR* **Abstract**: Lysergide *OR* **Abstract**: Lysergic Acid Diethylamide Tartrate *OR* **Abstract**: Mescaline *OR* **Abstract**: Mezcalin *OR* **Abstract**: Peyote *OR* **Abstract**: Trimethoxyphenethylamine *OR* **Abstract**: 3,4,5-Trimethoxyphenethylamine *AND* **Methodology**: Clinical Trial *AND* **Year**: 2013 *To* 2024

**Scopus:**

TITLE-ABS-KEY ( "Psilocybin" OR "Psilocybine" OR "DMT" OR "N Methyl 3,4 methylenedioxyamphetamine" OR "MDMA" OR "Methylenedioxymethamphetamine" OR "Ecstasy" OR "N-Methyl-3,4-methylenedioxyamphetamine Hydrochloride" OR "N Methyl 3,4 methylenedioxyamphetamine Hydrochloride" OR "N,N-Dimethyltryptamine" OR "Ayahuascas" OR "Banisteriopses" OR "Banisteriopsis" OR "Ayahuasca" OR "LSD" OR "LSD-25" OR "LSD 25" OR "Lysergic Acid Diethylamide" OR "Acid Diethylamide, Lysergic" OR "Diethylamide, Lysergic Acid" OR "Lysergide" OR "Lysergic Acid Diethylamide Tartrate" OR "Mescaline" OR "Mezcalin" OR "Peyote" OR "Trimethoxyphenethylamine" OR "3,4,5-Trimethoxyphenethylamine" ) AND TITLE-ABS-KEY ( "clinical trial" ) AND PUBYEAR > 2012 AND PUBYEAR < 2025

*Time filter publication was January 2013 to May 2024.

**Cochrane**:

(psilocybin or psilocybine or DMT or "N Methyl 3,4 methylenedioxyamphetamine" or MDMA or methylenedioxymethamphetamine or ecstasy or "N-Methyl-3,4-methylenedioxyamphetamine Hydrochloride" or "Hydrochloride, N-Methyl-3,4-methylenedioxyamphetamine" or "N Methyl 3,4 methylenedioxyamphetamine Hydrochloride" or "N,N-Dimethyltryptamine" or ayahuascas or banisteriopses or banisteriopsis or ayahuasca or LSD or LSD-25 or "LSD 25" or "Lysergic Acid Diethylamide" or "Acid Diethylamide, Lysergic" or "Diethylamide, Lysergic Acid" or lysergide or "Lysergic Acid Diethylamide Tartrate" or mescaline or mezcalin or peyote or trimethoxyphenethylamine or "3,4,5-Trimethoxyphenethylamine"):ti,ab,kw" with Publication Year from 2013 to 2024, in Trials

*Time filter publication was January 2013 to May 2024.
