# Supplementary material for: Music Playlist Use in Clinical Trials of Psychedelic Assisted Psychotherapy: A Systematic Review
Source: J Clin Psychol. 2026 Jun 15;82(9):1263–70. doi: 10.1002/jclp.70167 (PMC13441328; doi:10.1002/jclp.70167)
Supplement: Supplementary file 3 [file JCLP-82-1263-s003.docx]

**Articles and music playlist information source**

| Article | **Information source** |
| --- | --- |
| Percentage of Heavy Drinking Days Following Psilocybin-Assisted Psychotherapy vs Placebo in the Treatment of Adult Patients With Alcohol Use Disorder A Randomized Clinical Trial | Manuscript |
| Trial of Psilocybin versus Escitalopram for Depression. | Manuscript |
| Psilocybin with psychological support for treatment-resistant depression: an open-label feasibility study. | Manuscript |
| Reduction in social anxiety after MDMA-assisted psychotherapy with autistic adults: a randomized, double-blind, placebo-controlled pilot study. | Manuscript |
| Effects of Psilocybin-Assisted Therapy on Major Depressive Disorder: A Randomized Clinical Trial. | Manuscript |
| Increased global integration in the brain after psilocybin therapy for depression. | Manuscript |
| Ayahuasca Improves Self-perception of Speech Performance in Subjects With Social Anxiety Disorder: A Pilot, Proof-of-Concept, Randomized, Placebo-Controlled Trial. | Explicitly says that music was not allowed so there was no need to send and email |
| Safety and efficacy of lysergic acid diethylamide-assisted psychotherapy for anxiety associated with life-threatening diseases. | Manuscript |
| Single-Dose Psilocybin for a Treatment-Resistant Episode of Major Depression | Manuscript + Email |
| Psilocybin produces substantial and sustained decreases in depression and anxiety in patients with life-threatening cancer: A randomized double-blind trial | Manuscript |
| Lysergic Acid Diethylamide-Assisted Therapy in Patients With Anxiety With and Without a Life-Threatening Illness: A Randomized, Double-Blind, Placebo-Controlled Phase II Study. | Manuscript |
| 3,4-methylenedioxymethamphetamine (MDMA)-assisted psychotherapy for victims of sexual abuse with severe post-traumatic stress disorder: an open label pilot study in Brazil | Manuscript |
| Pilot study of the 5-HT2AR agonist psilocybin in the treatment of tobacco addiction. | Manuscript |
| MDMA-assisted therapy for severe PTSD: a randomized, double-blind, placebo-controlled phase 3 study. | Manuscript |
| 3,4-methylenedioxymethamphetamine (MDMA)-assisted psychotherapy for post-traumatic stress disorder in military veterans, firefighters, and police officers: a randomised, double-blind, dose-response, phase 2 clinical trial | Manuscript |
| MDMA-facilitated cognitive-behavioural conjoint therapy for posttraumatic stress disorder: An uncontrolled trial. | Manuscript |
| Therapeutic Alliance and Rapport Modulate Responses to Psilocybin Assisted Therapy for Depression | Manuscript |
| A randomized, controlled pilot study of MDMA (+/- 3,4-Methylenedioxymethamphetamine)-assisted psychotherapy for treatment of resistant, chronic Post-Traumatic Stress Disorder (PTSD) | Manuscript + Email |
| 3,4-Methylenedioxymethamphetamine-assisted psychotherapy for treatment of chronic posttraumatic stress disorder: A randomized phase 2 controlled trial. | Manuscript + Email |
| Rapid antidepressant effects of the psychedelic ayahuasca in treatment-resistant depression: a randomized placebo-controlled trial | Manuscript + Email |
| Rapid and sustained symptom reduction following psilocybin treatment for anxiety and depression in patients with life-threatening cancer: a randomized controlled trial. | Manuscript |
| Antidepressant Effects of a Single Dose of Ayahuasca in Patients With Recurrent Depression: A SPECT Study | Manuscript |
| Psilocybin-assisted therapy improves psycho-social-spiritual well-being in cancer patients | Manuscript + Email |
| Single-dose psilocybin-assisted therapy in major depressive disorder: a placebo-controlled, double-blind, randomised clinical trial | Manuscript |
| Scaling Up: Multisite Open-Label Clinical Trials of MDMA-Assisted Therapy for Severe Posttraumatic Stress Disorder | Manuscript |
| MDMA-assisted psychotherapy for treatment of anxiety and other psychological distress related to life-threatening illnesses: a randomized pilot study | Manuscript |
| Psilocybin for treatment resistant depression in patients taking a concomitant SSRI medication | Manuscript |
| MDMA-assisted therapy for moderate to severe PTSD: a randomized, placebo-controlled phase 3 trial | Manuscript |
| Psilocybin therapy for females with anorexia nervosa: a phase 1, open-label feasibility study | Manuscript |
| Pilot study of single-dose psilocybin for serotonin reuptake inhibitor-resistant body dysmorphic disorder | Manuscript |
| Psilocybin-assisted therapy for major depressive disorder: An exploratory placebo-controlled, fixed-order trial | Manuscript |
| Preliminary evidence for the importance of therapeutic alliance in MDMAassisted psychotherapy for posttraumatic stress disorder | Manuscript |
